# A force awakens: exploiting solar energy beyond photosynthesis

**DOI:** 10.1093/jxb/erz054

**Published:** 2019-02-18

**Authors:** David A Russo, Julie A Z Zedler, Poul Erik Jensen

**Affiliations:** Copenhagen Plant Science Centre, Department of Plant and Environmental Sciences, University of Copenhagen, Frederiksberg C, Denmark

**Keywords:** Cytochrome P450, light-driven catalysis, light-driven synthesis, photosynthetic chassis, photosensitizer, redox enzymes

## Abstract

In recent years, efforts to exploit sunlight, a free and abundant energy source, have sped up dramatically. Oxygenic photosynthetic organisms, such as higher plants, algae, and cyanobacteria, can convert solar energy into chemical energy very efficiently using water as an electron donor. By providing organic building blocks for life in this way, photosynthesis is undoubtedly one of the most important processes on Earth. The aim of light-driven catalysis is to harness solar energy, in the form of reducing power, to drive enzymatic reactions requiring electrons for their catalytic cycle. Light-driven enzymes have been shown to have a large number of biotechnological applications, ranging from the production of high-value secondary metabolites to the development of green chemistry processes. Here, we highlight recent key developments in the field of light-driven catalysis using biological components. We will also discuss strategies to design and optimize light-driven systems in order to develop the next generation of sustainable solutions in biotechnology.

## Introduction

Every ecosystem and food chain is dependent on primary producers enabling the fixation of inorganic CO_2_ into organic carbon skeletons. One possible route to achieve this is through the light reactions of photosynthesis, which facilitate the conversion of solar energy into chemical energy. The initial, light-dependent part of photosynthesis is a highly efficient process; however, the overall efficiency of photosynthesis is limited by the carbon-fixing enzyme ribulose-1,5-bisphosphate carboxylase/oxygenase. This results in a significant loss of energy as heat, fluorescence, and through several alternative electron sinks (Rochaix, 2011).

Global challenges such as rising CO_2_ levels and increasing temperatures require urgent action and novel solutions. In this context, biological platforms for the renewable production of chemical compounds and natural products, independent of petrochemistry, are gaining increasing interest. Given that sunlight is the only source of energy that is widely abundant on Earth and cost free, photosynthetic organisms are an obvious and promising choice for future production systems. These organisms are able to grow on CO_2_ as a carbon source, light as an energy source, and a minimal amount of inorganic nutrients.

Recently, efforts have been made to harness light beyond photosynthesis to directly power enzymes. This concept is often referred to as ‘light-driven catalysis’ (Box 1). In this article, we will give an overview of the state of the art in developing light-driven systems and discuss the most recent progress for high-value and commodity products. A short summary of recent key developments is given in Box 2. We will also expand on the current limitations for light-driven catalysis and demonstrate how the research community can address these limitations to make light-driven systems a major player in future sustainable biological production.

Box 1. **What is light-driven catalysis?**

The concept of light-driven catalysis refers to the direct supply of electrons, from a photosynthetic electron transport chain or a photosensitizer, to an enzyme for product formation. The electron transfer is driven by light, circumventing the need for cofactors that are in intracellular shortage, such as NAD(P)H. Electrons can be supplied to the target enzyme by (A) a photosynthetic electron transport chain (PETC) mediated by photosystem I (PSI) and ferredoxin, e.g. a cytochrome P450, (B) an internal cofactor that captures light, e.g. a fatty acid photodecarboxylase (FAP), (C) an excited photosensitizer, e.g. a lytic polysaccharide monooxygenase (LPMO), or (D) a PETC mediated by the plastoquinone (PQ) pool, e.g. a particulate methane monooxygenase (pMMO).

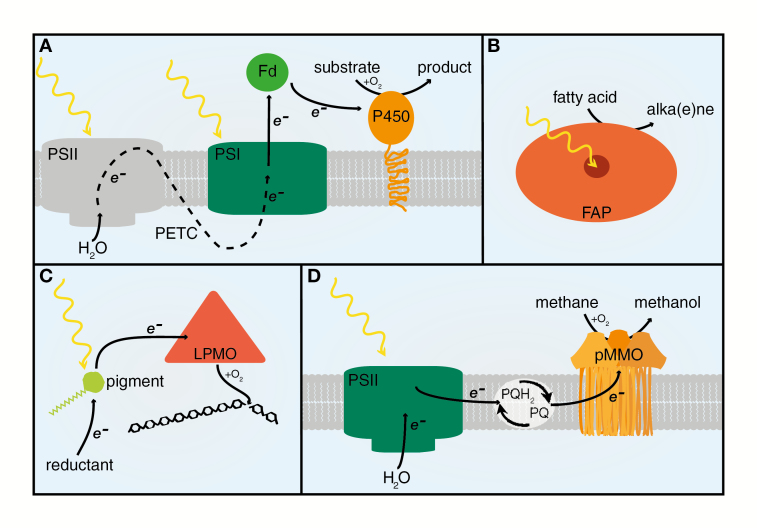

Box 2. **Key developments in light-driven catalysis**

- **Heterologous cytochrome P450 pathways can be driven by light in cyanobacteria**
Wlodarczyk *et al.* (2016) show that the dhurrin pathway can be expressed in a synthetic operon in *Synechocystis* sp. PCC 6803. The P450s successfully receive electrons from PSI and ferredoxin, thus enabling enzyme catalysis, both in *vitro* and *in vivo*, in a light-dependent manner.
- **LPMOs driven by photoexcited pigments can significantly increase biomass conversion**
Cannella *et al.* (2016) demonstrate light-induced polysaccharide oxidation using a combination of enzyme, pigment (photosensitizer), and reductant. This light-driven system increased polysaccharide degradation up to 100-fold.
- **A novel FAP converts long-chain fatty acids to alka(e)nes in microalgae**
Sorigué *et al.* (2017) describe an alka(e)ne-forming, blue-light-dependent photoenzyme in *Chlorella variabilis* and *Chlamydomonas reinhardtii*.
- **Demonstration of light-driven conversion of CO_2_ to hydrocarbons in microbial photosynthetic hosts**
  In a proof-of-concept study, Yunus *et al*, (2018) show light-driven hydrocarbon formation in *Synechocystis* sp. with the heterologous expression of the *Pseudomonas* fatty acid desaturase UndB and the *C. variabilis* FAP.
- **Light-driven hydroxylation of methane is established in methanotroph membranes**
Ito *et al.* (2018) demonstrate that light-driven catalysis can be established in non-photosynthetic membranes. A heterologous PSII was inserted in the membrane of *Methylosinus trichosporium* OB3b and used to drive the native membrane-bound pMMO.

## Light-driven synthesis of high-value compounds via photosystem I

### Cytochrome P450s are key enzymes of secondary metabolite synthesis

Higher plants produce a vast number of various specialized metabolites, such as terpenoids, cyanogenic glucosides, alkaloids, phenylpropanoids, and related phenolic compounds. The biosynthesis of these compounds requires highly specific and complex enzymatic reactions that often involve a group of monooxygenases named cytochrome P450s (P450s). P450s constitute one of the largest superfamilies of enzymes found in all biological kingdoms (Omura, 1999). P450s contain a heme prosthetic group, which mediates catalytic activation of oxygen under the use of electrons from NAD(P)H. Eukaryotic P450s are membrane bound, mainly to the endoplasmic reticulum (class II), but also to the mitochondrial and plastid membranes (class I), through an N-terminal transmembrane spanning segment (Schuler *et al.*, 2006; Bak *et al.*, 2011; Miyazaki *et al.*, 2011). Owing to their involvement in a plethora of plant biosynthetic pathways, the successful expression of highly active P450s is a crucial step in establishing the production of high-value compounds such as plant secondary metabolites.

### Heterologous expression of P450s in photosynthetic organisms for light-driven catalysis

The heterologous expression of class II eukaryotic P450s is a complex engineering exercise that requires attention to detail such as successful membrane integration and insertion of the heme group. Additionally, these P450s need a dedicated cytochrome P450 oxidoreductase that transfers electrons, via a flavin mononucleotide domain, from NADPH. Electron supply is generally considered to be the major limiting factor in native P450 systems (Jung *et al.*, 2011; Urlacher and Girhard, 2012). In order to overcome this limitation, it was proposed that, by introducing P450-containing pathways of interest into the plant chloroplast, these enzymes could use the surplus of reducing power generated by photosynthesis (Jensen *et al.*, 2011, 2012).

This concept was then demonstrated by the transient expression of two membrane-bound P450s (CYP79A1 and CYP71E1) and a soluble glycosyltransferase (UGT85B1) in *Nicotiana benthamiana*. The P450s were successfully inserted into the photosynthetic membranes, and *in vivo* activity was NADPH independent and mediated by the small 10–12 kDa soluble photoreduced electron carrier protein ferredoxin (Fd), thereby avoiding the need for a dedicated reductase (Nielsen *et al.*, 2013) (Box 1A). This was the first proof of *in vivo* light-driven catalysis; since then, it has been demonstrated with other pathways (Gnanasekaran *et al.*, 2015) and expression systems (Gnanasekaran *et al.*, 2016; Wlodarczyk *et al.*, 2016). Additionally, several individual P450s have been shown to be light-driven in photosynthetic hosts (Lassen *et al.*, 2014; Gangl *et al.*, 2015; Berepiki *et al.*, 2016; Zedler *et al.*, 2016; Berepiki *et al.*, 2018).

### Improving light-driven P450 systems by fusing dedicated electron carriers

A major limitation in powering light-driven systems with Fd is the competition with other, native, electron acceptors (Mellor *et al.*, 2017). In an attempt to make electron transfer more targeted and specific, Mellor *et al.* (2016) constructed synthetic P450-Fd fusion proteins using the P450 CYP79A1 as a model protein. The Fd part of the fusion protein could capture electrons directly from photosystem I (PSI), allowing the P450 to better compete with other electron sinks coupled to endogenous metabolic pathways. This study was able to show that the CYP79A1-Fd fusion enzyme obtained reducing power solely from its fused Fd and outperformed the unfused P450 *in vivo* (Mellor *et al.*, 2016; Mellor *et al.*, 2017).

### What is the next step for light-driven P450s?

We believe that, in particular, the production of plant-derived terpenoids will benefit from advances in light-driven P450 systems. These compounds are a large class of natural plant products with diverse applications (Bohlmann and Keeling, 2008). The biosynthesis of these molecules typically involves P450s functionalizing carbon backbones generated by terpene synthases (Pateraki *et al.*, 2015). The ample amount of precursor carbon skeletons, in combination with light-driven P450s, make photosynthetic organisms a very attractive choice as a terpenoid production chassis. It is encouraging to see that in recent years the research community has put increasing efforts into developing terpenoid production platforms in a range of photosynthetic, mainly microbial, hosts (Chaves and Melis, 2018; Knudsen *et al.*, 2018; Ko *et al.*, 2019; Lauersen, 2019; Lin and Pakrasi, 2019). More work is needed to determine whether the existing technology is scalable in a sustainable way.

## Redirecting photosynthetic reducing power into the production of drop-in fuels and commodities

Phototrophic production of biofuels has been stalled by limited production yields and technological difficulties (Jørgensen *et al.*, 2007; Reijnders, 2018). Therefore, recent efforts have combined design principles from synthetic biology and light-driven catalysis to engineer photosynthetic microbial hosts as chassis for the photobiological production of ‘drop-in compatible’ fuels (Aro, 2016). In photosynthetic microbes, the surplus of redox power (i.e. electrons) derived from the oxidation of water facilitates direct *in vivo* CO_2_-to-product conversion. Thus, these microbial chassis act as whole-cell biocatalysts capable of producing fuels and renewable chemicals compatible with existing value chains (Lin and Tao, 2017).

### Harnessing native pathways

The microalga *Botryococcus braunii* has a remarkable ability to naturally produce and secrete long-chain hydrocarbons (Metzger and Largeau, 2005). Despite the enormous potential of this organism, its slow growth and limited knowledge on its genome and physiology have precluded its industrial exploitation for hydrocarbon production (Cook *et al.*, 2017). Mining the *B. braunii* genome for novel enzymes and hydrocarbon partitioning and secretion routes that can be transferred into more tractable phototrophs might open up interesting opportunities. Sorigué and colleagues have published two important studies that broke new ground in eukaryotic hydrocarbon production. First, Sorigué *et al.* (2016) provided evidence that several green microalgal species (*Chlamydomonas reinhardtii*, *Chlorella variabilis* NC64A, and *Nannochloropsis* spp.) produce hydrocarbons in a light-dependent, but photosynthesis-independent, manner. A follow-up study identified the responsible enzyme, a fatty acid photodecarboxylase (FAP), which carries a flavin adenine dinucleotide as a light‐absorbing cofactor and converts fatty acids to hydrocarbons, with a quantum yield exceeding 0.8 (Sorigué *et al.*, 2017) (Box 1B).

Light-driven hydrocarbon production has also been found to occur in cyanobacteria. A novel pathway was shown to utilize Fd and Fd-NADP^+^ reductase to catalyse the conversion of acyl–acyl carrier proteins (acyl-ACP) to alkanes (Schirmer *et al.*, 2010; Zhang *et al.*, 2013). These enzymes were named acyl-ACP reductase (AAR) and aldehyde decarbonylase [later renamed to aldehyde-deformylating oxygenase (ADO); Li *et al.*, 2012]. Interestingly, orthologs of both enzymes were identified in 90% of cyanobacterial genomes, suggesting an unexpected ubiquity of this pathway (Klähn *et al.*, 2014). To date, attempts to engineer the native AAR/ADO pathway have not resulted in significant improvements (Wang *et al.*, 2013; Peramuna *et al.*, 2015; Yunus *et al.*, 2018). This suggests a high degree of native pathway regulation. Further research is required to elucidate the intricacies of the cyanobacterial AAR/ADO pathway.

### Engineering *de novo* pathways

In a recent study, Yunus *et al.* (2018) demonstrated the potential of photosynthetic microorganisms for heterologous production of drop-in compatible biofuels. This work describes a side-by-side comparison of a thioesterase (TesA)/Δ*aas Synechocystis* sp. PCC 6803 strain and a TesA *C. reinhardtii* mutant, both engineered with a series of synthetic pathways for hydrocarbon production (Yunus *et al.*, 2018). In *C. reinhardtii*, TesA was unable to increase the free fatty acid (FFA) pool, thus hindering further attempts to increase hydrocarbon production. On the other hand, in *Synechocystis*, FFAs were efficiently liberated and two distinct light-driven pathways were introduced. First, the *Pseudomonas* aldehyde decarboxylase UndB was expressed and converted 55% of liberated FFAs into their respective alkenes. In its native environment, UndB is an integral membrane protein and requires electrons for its catalytic cycle (Rui *et al.*, 2015). These observations allow us to speculate that UndB might be inserted into the thylakoid membranes and could potentially be light-driven. The source of the UndB electron supply in the study was, however, unknown (Yunus *et al.*, 2018). Second, the *C. variabilis* FAP was introduced into *Synechocystis* and produced up to 77.1 mg g^–1^ cell dry weight of alkanes (a 9-fold increase over a *C. reinhardtii* FAP overexpresser strain; Yunus *et al.*, 2018). This suggests that, in certain cases, heterologous expression of a target enzyme may be preferable to native pathway manipulation. Despite the successful reconstitution of heterologous hydrocarbon pathways, both strains suffered from impaired growth due to intracellular accumulation of products (Yunus *et al.*, 2018). Therefore, understanding and controlling product secretion is crucial for improvement of *in vivo* hydrocarbon production.

## *In vitro* systems for light-driven catalysis

Many light-driven enzymes are natively membrane-bound (e.g. P450s); therefore, their large-scale application in an industrial setting is challenging. This issue was addressed in a recent study seeking to improve the application of particulate methane monooxygenases (pMMOs) (Ito *et al.*, 2018). These membrane-bound metalloproteins require two electrons to oxidize the C-H bond in methane to form methanol (Basch *et al.*, 1999). The conversion of methane to methanol under mild conditions, for example, using enzymatic conversion, is a highly sought after reaction. It has the potential to simplify the transport and storage of methane and expand the portfolio of natural gas-based fuels. Ito *et al.* (2018) reconstituted photosystem II (PSII) into the pMMO-containing membrane fraction of *Methylosinus trichosporium* OB3d. This created a sequential redox chain where the pMMO was driven by membrane-bound quinones reduced by electrons originating from light-driven oxidation of water (Box 1D). However, light intensities above 20 µmol m^−2^ s^−1^ led to PSII decomposition and, consequentially, to lower activity levels. In order to generate a more robust system, it would be of interest to design a system with the pMMO inserted into a photosynthetic membrane. However, the complexity of the enzyme (a homotrimer with three distinct metal centres and seven transmembrane domains; Liebeman and Rosenzweig, 2005) might constitute a major engineering challenge.

In cases of enzymes that are soluble and stable in physiological conditions, their large-scale application is less complex. For example, a recent study demonstrated this by developing a light-driven system with a lytic polysaccharide monooxygenase (LPMO) (Box 1C) (Cannella *et al.*, 2016). LPMOs are copper metalloproteins that require electrons and O_2_ or H_2_O_2_ to disrupt polysaccharide chains (Frandsen and Lo Leggio, 2016; Frommhagen *et al.*, 2018; Tandrup *et al.*, 2018). Most often, electrons are supplied by small molecule reductants (e.g. ascorbic acid). However, Cannella *et al.* (2016) demonstrated that a combination of a photosensitizer (chlorophyllin/thylakoids) and a reductant (ascorbic acid), under low-intensity visible light exposure (150–200 µmol m^−2^ s^−1^), increased the catalytic activity of the LPMO 10- to 100-fold. While the mechanistic details of this light-driven system are still under intense debate (Möllers *et al.*, 2017; Bissaro *et al.*, 2018), the commercial potential of this system is unquestionable.

## Light-driven catalysis: where do we go from here?

The concept of light-driven catalysis is still in its infancy; however, the developments highlighted here show that it is maturing quickly. Moving forward, we should utilize the biomimetic principle of ‘biology to design’ to learn from native systems in order to improve upon existing light-driven solutions. With that in mind, we would like to highlight two key considerations when designing a light-driven system: (i) the choice of an appropriate host chassis and (ii) the exploitation of synthetic biology to rationally design and organize future light-driven systems.

### Choosing an appropriate host chassis for a specific light-driven system

A consistent supply of electrons is required for an efficient light-driven system. Consequently, any organism capable of performing photosynthesis will be a superior choice of host chassis compared with traditional heterotrophic microbial systems. Additionally, there are several general aspects that are desirable in any production host: fast growth, efficient transformation systems, and the availability of tools to design and regulate light-driven catalysis.

For example, in plants, transient expression can yield very high levels of recombinant protein, making it an attractive choice (Sainsbury and Lomonossoff, 2014); this is particularly the case for *N. benthamiana* (Bally *et al.*, 2018). However, engineering tools to directly modify plant chloroplasts, the location of light-driven catalysis, and generate stable transformants are still lagging behind those for unicellular organisms (Mellor *et al.*, 2018). On the other hand, in cyanobacteria, engineering strategies are relatively advanced, with a large availability of different tools (Klemenčič *et al.*, 2017; Sengupta *et al*., 2018), and several species have already been shown to be suitable for the integration of light-driven systems (Lassen *et al.*, 2014; Wlodarczyk *et al.*, 2016). Furthermore, there have also been rapid developments in identifying and characterizing fast-growing cyanobacterial species (Yu *et al.*, 2015; Ungerer *et al.*, 2018) that are key to making photosynthetic chassis more competitive.

Overall, the particular choice of host organism will have to be determined based on the individual product of interest (considering, for example, the metabolic architecture and availability of precursors), which might require testing different photosynthetic chassis to find the most suited one. In this context, it is encouraging that an array of standardized DNA assembly systems is becoming available for photosynthetic hosts. One example is the Golden Gate-based methods developed for both plants and *C. reinhardtii* (Engler *et al.*, 2009, 2014; Crozet *et al.*, 2018), thus also facilitating easier exchange of parts between different host chassis. Additionally, the emergence of CRISPR/Cas nuclease gene-editing technology in a wide range of organisms has provided the tools to engineer host chassis with unprecedented precision (Doudna and Charpentier, 2014).

### Synthetic biology is key to the advancement of light-driven systems

**Fig. 1.**
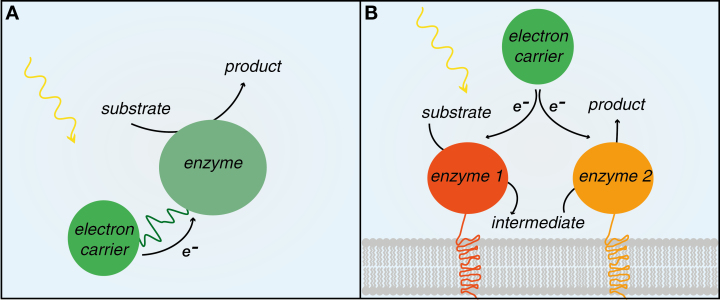
Key strategies to improve light-driven systems. (A) Fusion of target enzyme and electron carrier. (B) Spatial organization (co-localization) of individual components, in this case shown by tethering to the membrane.

The road to the industrial use of light-driven systems contains numerous engineering and optimization challenges. We believe that key strategies to overcome these challenges can be implemented with synthetic biology (Fig. 1).

In a light-driven system, photosynthetic reducing power is abundant; however, competition from native electron transfer pathways and the inherent regulation of light-harvesting and photosynthetic electron transport may constitute obstacles. Therefore, to take full advantage of the electron flux, it is advisable to design a system where the electron donor and target enzyme present a suitable redox coupling while also considering potential off-target redox partners (Mellor *et al.*, 2017). To improve electron channelling, fusing an electron carrier to the target enzyme has been shown to improve catalysis (Mellor *et al.*, 2016) (Fig. 1A). Going forward, considering other electron carriers and controlling tight interactions between electron carrier and enzyme are very likely to improve fluxes of light-driven systems. Thus, the design of a suitable redox cascade between interaction partners in a light-driven system should be a major design consideration, and an array of suitable candidates, with defined midpoint potentials, should be developed as standardized, exchangeable parts.

Another strategy to achieve more efficient and controlled interactions within a light-driven system is to spatially organize individual components (Fig. 1B). Synthetic biology offers a seemingly unlimited number of options to achieve this. A recent study showed that the individual enzymes of the dhurrin pathway can be transiently expressed in *N. tabacum* with membrane anchors from components of the twin-arginine protein translocation system, which natively co-localize and assemble in the thylakoid membrane. This strategy increased the final dhurrin yield 5-fold (Henriques de Jesus *et al.*, 2017). This study demonstrates how controlling the localization of heterologous enzymes can improve light-driven systems. Moreover, this is a first example of using enzyme scaffolding to build light-driven metabolons within the native cell architecture. Synthetic biology is, however, not limited to targeting the native host cell architecture, but can also be used to design entirely synthetic scaffolds and even compartments, as recently shown in yeast (Lau *et al.*, 2018). Utilizing synthetic structures will allow the design of dedicated sites for light-driven catalysis that are controllable and free of endogenous regulation. Despite the promising strategies outlined above, their application requires a detailed understanding of protein trafficking and targeting within the cell. Additionally, the localization of not only the enzymes needs to be controlled, but also that of the final product: can the product be secreted into the medium, or can it be stored in a subcellular compartment? Again, a detailed understanding of the host chassis metabolism and cellular organization is necessary.

Overall, combining the key strategies outlined above should provide new avenues to design and fine-tune the next generation of light-driven enzymes and biosynthetic pathways. Together with a more sophisticated understanding of metabolic fluxes and trade-offs between growth and production (Knoop and Steuer, 2015), light-driven catalysis can play a major role in exploiting solar energy beyond photosynthesis.

## References

[CIT0001] Aro EM 2016 From first generation biofuels to advanced solar biofuels. Ambio 45(Suppl 1), S24–S31. 26667057 10.1007/s13280-015-0730-0 PMC4678123

[CIT0002] Bak S, Beisson F, Bishop G, Hamberger B, Höfer R, Paquette S, Werck-Reichhart D 2011 Cytochromes p450. The Arabidopsis Book 9, e0144. 22303269 10.1199/tab.0144 PMC3268508

[CIT0003] Bally J, Jung H, Mortimer C, Naim F, Philips JG, Hellens R, Bombarely A, Goodin MM, Waterhouse PM 2018 The rise and rise of *Nicotiana benthamiana*: a plant for all reasons. Annual Review of Phytopathology 56, 405–426. 10.1146/annurev-phyto-080417-050141 30149789

[CIT0004] Basch H, Mogi K, Musaev DG, Morokuma K 1999 Mechanism of the methane → methanol conversion reaction catalyzed by methane monooxygenase: a density functional study. Journal of the American Chemical Society 121, 7249–7256.

[CIT0005] Berepiki A, Gittins JR, Moore CM, Bibby TS 2018 Rational engineering of photosynthetic electron flux enhances light-powered cytochrome P450 activity. Synthetic Biology 3, ysy009. 10.1093/synbio/ysy009 PMC7445785 32995517

[CIT0006] Berepiki A, Hitchcock A, Moore CM, Bibby TS 2016 Tapping the unused potential of photosynthesis with a heterologous electron sink. ACS Synthetic Biology 5, 1369–1375. 27437951 10.1021/acssynbio.6b00100

[CIT0007] Bissaro B, Várnai A, Røhr ÅK, Eijsink VGH 2018 Oxidoreductases and reactive oxygen species in conversion of lignocellulosic biomass. Microbiology and Molecular Biology Reviews 82, e00029-18. 30257993 10.1128/MMBR.00029-18 PMC6298611

[CIT0008] Bohlmann J, Keeling CI 2008 Terpenoid biomaterials. The Plant Journal 54, 656–669. 18476870 10.1111/j.1365-313X.2008.03449.x

[CIT0009] Cannella D, Möllers KB, Frigaard NU, Jensen PE, Bjerrum MJ, Johansen KS, Felby C 2016 Light-driven oxidation of polysaccharides by photosynthetic pigments and a metalloenzyme. Nature Communications 7, 11134. 10.1038/ncomms11134 PMC4822002 27041218

[CIT0010] Chaves JE, Melis A 2018 Engineering isoprene synthesis in cyanobacteria. FEBS Letters 592, 2059–2069. 29689603 10.1002/1873-3468.13052

[CIT0011] Cook C, Dayananda C, Tennant RK, Love J 2017 Third-generation biofuels from the microalga, *Botryococcus braunii.* In: Love J, Bryant JA, eds. Biofuels and bioenergy. Chichester: John Wiley & Sons Ltd, 157–171.

[CIT0012] Crozet P, Navarro FJ, Willmund F, et al 2018 Birth of a photosynthetic chassis: a MoClo toolkit enabling synthetic biology in the microalga *Chlamydomonas reinhardtii*. ACS Synthetic Biology 7, 2074–2086. 30165733 10.1021/acssynbio.8b00251

[CIT0013] Doudna JA, Charpentier E 2014 Genome editing. The new frontier of genome engineering with CRISPR-Cas9. Science 346, 1258096. 25430774 10.1126/science.1258096

[CIT0014] Engler C, Gruetzner R, Kandzia R, Marillonnet S 2009 Golden gate shuffling: a one-pot DNA shuffling method based on type IIs restriction enzymes. PLoS ONE 4, e5553. 19436741 10.1371/journal.pone.0005553 PMC2677662

[CIT0015] Engler C, Youles M, Gruetzner R, Ehnert TM, Werner S, Jones JD, Patron NJ, Marillonnet S 2014 A golden gate modular cloning toolbox for plants. ACS Synthetic Biology 3, 839–843. 24933124 10.1021/sb4001504

[CIT0016] Frandsen KE, Lo Leggio L 2016 Lytic polysaccharide monooxygenases: a crystallographer’s view on a new class of biomass-degrading enzymes. IUCrJ 3, 448–467. 10.1107/S2052252516014147 PMC5094447 27840684

[CIT0017] Frommhagen M, Westphal AH, van Berkel WJH, Kabel MA 2018 Distinct substrate specificities and electron-donating systems of fungal lytic polysaccharide monooxygenases. Frontiers in Microbiology 9, 1080. 29896168 10.3389/fmicb.2018.01080 PMC5987398

[CIT0018] Gangl D, Zedler JAZ, Włodarczyk A, Jensen PE, Purton S, Robinson C 2015 Expression and membrane-targeting of an active plant cytochrome P450 in the chloroplast of the green alga *Chlamydomonas reinhardtii*. Phytochemistry 110, 22–28. 25556316 10.1016/j.phytochem.2014.12.006

[CIT0019] Gnanasekaran T, Karcher D, Nielsen AZ, et al 2016 Transfer of the cytochrome P450-dependent dhurrin pathway from *Sorghum* bicolor *into Nicotiana tabacum* chloroplasts for light-driven synthesis. Journal of Experimental Botany 67, 2495–2506. 26969746 10.1093/jxb/erw067 PMC4809297

[CIT0020] Gnanasekaran T, Vavitsas K, Andersen-Ranberg J, Nielsen AZ, Olsen CE, Hamberger B, Jensen PE 2015 Heterologous expression of the isopimaric acid pathway in *Nicotiana benthamiana* and the effect of N-terminal modifications of the involved cytochrome P450 enzyme. Journal of Biological Engineering 9, 24. 26702299 10.1186/s13036-015-0022-z PMC4688937

[CIT0021] Henriques de Jesus MPR, Zygadlo Nielsen A, Busck Mellor S, Matthes A, Burow M, Robinson C, Jensen PE 2017 Tat proteins as novel thylakoid membrane anchors organize a biosynthetic pathway in chloroplasts and increase product yield 5-fold. Metabolic Engineering 44, 108–116. 28962875 10.1016/j.ymben.2017.09.014

[CIT0022] Ito H, Kondo R, Yoshimori K, Kamachi T 2018 Methane hydroxylation with water as an electron donor under light irradiation in the presence of reconstituted membranes containing both photosystem II and a methane monooxygenase. ChemBioChem 19, 2152–2155. 30246911 10.1002/cbic.201800324

[CIT0023] Jensen K, Jensen PE, Møller BL 2011 Light-driven cytochrome p450 hydroxylations. ACS Chemical Biology 6, 533–539. 21323388 10.1021/cb100393j

[CIT0024] Jensen K, Jensen PE, Møller BL 2012 Light-driven chemical synthesis. Trends in Plant Science 17, 60–63. 22306522 10.1016/j.tplants.2011.12.008

[CIT0025] Jørgensen H, Kristensen JB, Felby C 2007 Enzymatic conversion of lignocellulose into fermentable sugars: challenges and opportunities. Biofuels, Bioproducts and Biorefining 1, 119–134.

[CIT0026] Jung ST, Lauchli R, Arnold FH 2011 Cytochrome P450: taming a wild type enzyme. Current Opinion in Biotechnology 22, 809–817. 21411308 10.1016/j.copbio.2011.02.008 PMC3118264

[CIT0027] Klähn S, Baumgartner D, Pfreundt U, Voigt K, Schön V, Steglich C, Hess WR 2014 Alkane biosynthesis genes in cyanobacteria and their transcriptional organization. Frontiers in Bioengineering and Biotechnology 2, 24. 25022427 10.3389/fbioe.2014.00024 PMC4094844

[CIT0028] Klemenčič M, Nielsen AZ, Sakuragi Y, Frigaard NU, Čelešnik H, Jensen PE, Dolinar M 2017 Synthetic biology of cyanobacteria for production of biofuels and high-value products. In: Gonzalez-Fernandez C, Muñoz R, eds. Microalgae-based biofuels and bioproducts. Duxford: Woodhead Publishing, 305–325.

[CIT0029] Knoop H, Steuer R 2015 A computational analysis of stoichiometric constraints and trade-offs in cyanobacterial biofuel production. Frontiers in Bioengineering and Biotechnology 3, 47. 25941672 10.3389/fbioe.2015.00047 PMC4403605

[CIT0030] Knudsen C, Gallage NJ, Hansen CC, Møller BL, Laursen T 2018 Dynamic metabolic solutions to the sessile life style of plants. Natural Product Reports 35, 1140–1155. 30324199 10.1039/c8np00037a PMC6254060

[CIT0031] Ko SC, Lee HJ, Choi SY, Choi J-i, Woo HM 2019 Bio-solar cell factories for photosynthetic isoprenoids production. Planta 249, 181–193. 30078076 10.1007/s00425-018-2969-8

[CIT0032] Lassen LM, Nielsen AZ, Olsen CE, Bialek W, Jensen K, Møller BL, Jensen PE 2014 Anchoring a plant cytochrome P450 via PsaM to the thylakoids in *Synechococcus* sp. PCC 7002: evidence for light-driven biosynthesis. PLoS ONE 9, e102184. 25025215 10.1371/journal.pone.0102184 PMC4099078

[CIT0033] Lau YH, Giessen TW, Altenburg WJ, Silver PA 2018 Prokaryotic nanocompartments form synthetic organelles in a eukaryote. Nature Communications 9, 1311. 10.1038/s41467-018-03768-x PMC5882880 29615617

[CIT0034] Lauersen KJ 2019 Eukaryotic microalgae as hosts for light-driven heterologous isoprenoid production. Planta 249, 155–180. 30467629 10.1007/s00425-018-3048-x

[CIT0035] Li N, Chang WC, Warui DM, Booker SJ, Krebs C, Bollinger JM Jr 2012 Evidence for only oxygenative cleavage of aldehydes to alk(a/e)nes and formate by cyanobacterial aldehyde decarbonylases. Biochemistry 51, 7908–7916. 22947199 10.1021/bi300912n

[CIT0036] Lieberman RL, Rosenzweig AC 2005 Crystal structure of a membrane-bound metalloenzyme that catalyses the biological oxidation of methane. Nature 434, 177–182. 15674245 10.1038/nature03311

[CIT0037] Lin P-C, Pakrasi HB 2018 Engineering cyanobacteria for production of terpenoids. Planta 249, 145–154. 30465115 10.1007/s00425-018-3047-y

[CIT0038] Lin B, Tao Y 2017 Whole-cell biocatalysts by design. Microbial Cell Factories 16, 106. 28610636 10.1186/s12934-017-0724-7 PMC5470193

[CIT0039] Mellor SB, Behrendorff JBYH, Nielsen AZ, Jensen PE, Pribil M 2018 Non-photosynthetic plastids as hosts for metabolic engineering. Essays in Biochemistry 62, 41–50. 29487195 10.1042/EBC20170047

[CIT0040] Mellor SB, Nielsen AZ, Burow M, Motawia MS, Jakubauskas D, Møller BL, Jensen PE 2016 Fusion of ferredoxin and cytochrome P450 enables direct light-driven biosynthesis. ACS Chemical Biology 11, 1862–1869. 27119279 10.1021/acschembio.6b00190 PMC4949584

[CIT0041] Mellor SB, Vavitsas K, Nielsen AZ, Jensen PE 2017 Photosynthetic fuel for heterologous enzymes: the role of electron carrier proteins. Photosynthesis Research 134, 329–342. 28285375 10.1007/s11120-017-0364-0

[CIT0042] Metzger P, Largeau C 2005 *Botryococcus braunii*: a rich source for hydrocarbons and related ether lipids. Applied Microbiology and Biotechnology 66, 486–496. 15630516 10.1007/s00253-004-1779-z

[CIT0043] Miyazaki S, Katsumata T, Natsume M, Kawaide H 2011 The CYP701B1 of *Physcomitrella patens* is an ent-kaurene oxidase that resists inhibition by uniconazole-P. FEBS Letters 585, 1879–1883. 21545802 10.1016/j.febslet.2011.04.057

[CIT0044] Möllers KB, Mikkelsen H, Simonsen TI, Cannella D, Johansen KS, Bjerrum MJ, Felby C 2017 On the formation and role of reactive oxygen species in light-driven LPMO oxidation of phosphoric acid swollen cellulose. Carbohydrate Research 448, 182–186. 28335986 10.1016/j.carres.2017.03.013

[CIT0045] Nielsen AZ, Ziersen B, Jensen K, Lassen LM, Olsen CE, Møller BL, Jensen PE 2013 Redirecting photosynthetic reducing power toward bioactive natural product synthesis. ACS Synthetic Biology 2, 308–315. 23654276 10.1021/sb300128r

[CIT0046] Omura T 1999 Forty years of cytochrome P450. Biochemical and Biophysical Research Communications 266, 690–698. 10603307 10.1006/bbrc.1999.1887

[CIT0047] Pateraki I, Heskes AM, Hamberger B 2015 Cytochromes P450 for terpene functionalisation and metabolic engineering. In: Schrader J, Bohlmann J, eds. Biotechnology of isoprenoids. Cham: Springer International Publishing, 107–139. 10.1007/10_2014_301 25636487

[CIT0048] Peramuna A, Morton R, Summers ML 2015 Enhancing alkane production in cyanobacterial lipid droplets: a model platform for industrially relevant compound production. Life 5, 1111–1126. 25821934 10.3390/life5021111 PMC4500132

[CIT0049] Reijnders L 2018 Biofuels from microalgae: biodiesel. In: Jacob-Lopes E, Queiroz Zepka L, Queiroz MI, eds. Energy from microalgae. Cham: Springer International Publishing, 171–180.

[CIT0050] Rochaix JD 2011 Regulation of photosynthetic electron transport. Biochimica et Biophysica Acta 1807, 375–383. 21118674 10.1016/j.bbabio.2010.11.010

[CIT0051] Rui Z, Harris NC, Zhu X, Huang W, Zhang W 2015 Discovery of a family of desaturase-like enzymes for 1-alkene biosynthesis. ACS Catalysis 5, 7091–7094.

[CIT0052] Sainsbury F, Lomonossoff GP 2014 Transient expressions of synthetic biology in plants. Current Opinion in Plant Biology 19, 1–7. 24631883 10.1016/j.pbi.2014.02.003 PMC4070481

[CIT0053] Schirmer A, Rude MA, Li X, Popova E, del Cardayre SB 2010 Microbial biosynthesis of alkanes. Science 329, 559–562. 20671186 10.1126/science.1187936

[CIT0054] Schuler MA, Duan H, Bilgin M, Ali S 2006 *Arabidopsis* cytochrome P450s through the looking glass: a window on plant biochemistry. Phytochemistry Reviews 5, 205–237.

[CIT0055] Sengupta A, Pakrasi HB, Wangikar PP 2018 Recent advances in synthetic biology of cyanobacteria. Applied Microbiology and Biotechnology 102, 5457–5471. 29744631 10.1007/s00253-018-9046-x

[CIT0056] Sorigué D, Légeret B, Cuiné S, et al 2017 An algal photoenzyme converts fatty acids to hydrocarbons. Science 357, 903–907. 28860382 10.1126/science.aan6349

[CIT0057] Sorigué D, Légeret B, Cuiné S, Morales P, Mirabella B, Guédeney G, Li-Beisson Y, Jetter R, Peltier G, Beisson F 2016 Microalgae synthesize hydrocarbons from long-chain fatty acids via a light-dependent pathway. Plant Physiology 171, 2393–2405. 27288359 10.1104/pp.16.00462 PMC4972275

[CIT0058] Tandrup T, Frandsen KEH, Johansen KS, Berrin JG, Lo Leggio L 2018 Recent insights into lytic polysaccharide monooxygenases (LPMOs). Biochemical Society Transactions 46, 1431–1447. 30381341 10.1042/BST20170549

[CIT0059] Ungerer J, Wendt KE, Hendry JI, Maranas CD, Pakrasi HB 2018 Comparative genomics reveals the molecular determinants of rapid growth of the cyanobacterium *Synechococcus elongatus* UTEX 2973. Proceedings of the National Academy of Sciences, USA 115, E11761–E11770. 10.1073/pnas.1814912115 PMC6294925 30409802

[CIT0060] Urlacher VB, Girhard M 2012 Cytochrome P450 monooxygenases: an update on perspectives for synthetic application. Trends in Biotechnology 30, 26–36. 21782265 10.1016/j.tibtech.2011.06.012

[CIT0061] Wang W, Liu X, Lu X 2013 Engineering cyanobacteria to improve photosynthetic production of alka(e)nes. Biotechnology for Biofuels 6, 69. 23641684 10.1186/1754-6834-6-69 PMC3679977

[CIT0062] Wlodarczyk A, Gnanasekaran T, Nielsen AZ, et al 2016 Metabolic engineering of light-driven cytochrome P450 dependent pathways into *Synechocystis* sp. PCC 6803. Metabolic Engineering 33, 1–11. 26548317 10.1016/j.ymben.2015.10.009

[CIT0063] Yu J, Liberton M, Cliften PF, Head RD, Jacobs JM, Smith RD, Koppenaal DW, Brand JJ, Pakrasi HB 2015 *Synechococcus elongatus* UTEX 2973, a fast growing cyanobacterial chassis for biosynthesis using light and CO₂. Scientific Reports 5, 8132. 25633131 10.1038/srep08132 PMC5389031

[CIT0064] Yunus IS, Wichmann J, Wördenweber R, Lauersen KJ, Kruse O, Jones PR 2018 Synthetic metabolic pathways for photobiological conversion of CO_2_ into hydrocarbon fuel. Metabolic Engineering 49, 201–211. 30144559 10.1016/j.ymben.2018.08.008

[CIT0065] Zedler JAZ, Gangl D, Guerra T, Santos E, Verdelho VV, Robinson C 2016 Pilot-scale cultivation of wall-deficient transgenic *Chlamydomonas reinhardtii* strains expressing recombinant proteins in the chloroplast. Applied Microbiology and Biotechnology 100, 7061–7070. 26969037 10.1007/s00253-016-7430-y

[CIT0066] Zhang J, Lu X, Li JJ 2013 Conversion of fatty aldehydes into alk (a/e)nes by *in vitro* reconstituted cyanobacterial aldehyde-deformylating oxygenase with the cognate electron transfer system. Biotechnology for Biofuels 6, 86. 23759169 10.1186/1754-6834-6-86 PMC3691600

